# Chemistry, as Exemplifying the Wisdom and Beneficence of God; Being the Actonian Prize Essay

**Published:** 1844-10

**Authors:** 


ART. VII.—1. *Chemistry, as exemplifying the Wisdom and Beneficence of God; being the Actonian Prize Essay.* By GEORGE FOWNES, PH.D., Chemical Lecturer in the Middlesex Hospital Medical School.—*London*, 1844. Small 8vo, pp. 184.

2. *An Introduction to Practical Organic Chemistry; with references to the works of Davy, Brande, Liebig, &c.—London*, 1843. 12mo, pp. 90.

3. *The Chemical and Physiological Balance of Organic Nature; an Essay by* MM. DUMAS and BOUSSINGAULT, Members of the Institute of France. Third Edition, with New Documents.—*London*, 1844. 12mo, pp. 166.

OUR readers need not be told, that the recent progress of organic chemistry is rendering it quite the fashionable subject of the day; and so it will probably be, until the brilliance of novelty has faded, or a new discovery in some other branch of science has (to use a vulgar but very expressive phrase,) "taken the shine out of it;" or until it is found as we believe it shortly will be, that there are many things in it which are *new*, without being *true*. Nevertheless, we hold it incumbent upon every medical man to do his best to keep pace with the progress of the science; because, even in its present state, it is capable of affording to the intelligent practitioner many valuable hints in regard to the treatment of disease; and also because the public in general are now becoming so very learned on the subject, (thanks to the articles of Drs. Playfair and Gregory in the Edinburgh and Quarterly Review,) that the name of Liebig is becoming a household word, and the incense he receives is little less than that which was offered to the deities of old.—The first work on our list is not professedly a treatise on organic chemistry; but this department of the subject naturally occupies a large part of the author's attention. The sketch it contains of the present condition of the science, is extremely lucid and comprehensive; but we think that, as a treatise on natural theology, it is in many points deficient.—The second of these little volumes is the fourth of the series of 'Small Books on Great Subjects;' on some former parts of which we have heretofore bestowed our warm commendation. The present treatise fully bears out the character of its predecessors; being like them, a masterly outline of the subject it embraces, drawn with a vigorous hand, and a truly philosophic spirit, and developing the practical applications of the truths it enunciates, as well in regard to the arts of life, as with respect to the mental improvement of the cultivator of science.—The third is a translation of a discourse delivered by M. Dumas at the Ecole de Médecine; which is probably already familiar to most of our readers, through the pages of the British journals, into which it was transferred at the time of its first publication. It now appears in a separate form, with many additions, and with the results of new experiments. Although the discourse was delivered by M. Dumas, and passes by his name, he desires that it should be regarded as the expression of the views, at which M. Boussingault and himself have jointly arrived, from their united researches on this subject. It is pleasing to see that such harmony can exist between two labourers in the same field; and we could wish that it were more prevalent. In particular, we cannot but regret the schism which exists between the leaders of the French and of the German schools; each party accusing the other of having pirated their opinions. Whether M. Dumas, or M. Liebig, was the first to enunciate the doctrines in question, we shall not now attempt to determine; but this much is certain, that both have taken as their foundation the patient and laborious researches of M. Boussingault, to whom a very large share of the merit is therefore due. No student of organic chemistry should be without the last of these treatises; and to those who desire to acquire a general knowledge of what is being done in this department of science, we strongly recommend the perusal of either or both of the two former.

